# The gut microbiota drives the impact of bile acids and fat source in diet on mouse metabolism

**DOI:** 10.1186/s40168-018-0510-8

**Published:** 2018-08-02

**Authors:** Sarah Just, Stanislas Mondot, Josef Ecker, Katrin Wegner, Eva Rath, Laura Gau, Theresa Streidl, Genevieve Hery-Arnaud, Sinah Schmidt, Till Robin Lesker, Valentin Bieth, Andreas Dunkel, Till Strowig, Thomas Hofmann, Dirk Haller, Gerhard Liebisch, Philippe Gérard, Sascha Rohn, Patricia Lepage, Thomas Clavel

**Affiliations:** 1 ZIEL–Institute for Food and Health, Technical University of Munich, Freising, Germany; 2 Micalis Institute, INRA, AgroParisTech, University Paris-Saclay, Jouy-en-Josas, France; 3 Nutritional Physiology, Technical University of Munich, Freising, Germany; 4 Institute of Food Chemistry, Hamburg School of Food Science, University of Hamburg, Hamburg, Germany; 5 Nutrition and Immunology, Technical University of Munich, Freising, Germany; 6 Institute of Medical Microbiology, Functional Microbiome Research Group, University Hospital of RWTH Aachen, Pauwelsstrasse 30, 52074 Aachen, Germany; 7 Food Chemistry and Molecular and Sensory Science, Technical University of Munich, Freising, Germany; 8 Research Group Microbial Immune Regulation, Helmholtz Centre for Infection Research, Braunschweig, Germany; 9 Institute of Clinical Chemistry and Laboratory Medicine, University of Regensburg, Regensburg, Germany

**Keywords:** Metabolic diseases, Diet-induced obesity, Gut microbiota, Germ-free mice, Bile acids, Dietary fat, Lard, Lipidomics, 16S rRNA gene amplicon sequencing, Metatranscriptomics

## Abstract

**Background:**

As the gut microbiota contributes to metabolic health, it is important to determine specific diet-microbiota interactions that influence host metabolism. Bile acids and dietary fat source can alter phenotypes of diet-induced obesity, but the interplay with intestinal microorganisms is unclear. Here, we investigated metabolic consequences of diets enriched in primary bile acids with or without addition of lard or palm oil, and studied gut microbiota structure and functions in mice.

**Results:**

In combination with bile acids, dietary lard fed to male C57BL/6N mice for a period of 8 weeks enhanced fat mass accumulation in colonized, but not in germ-free mice when compared to palm oil. This was associated with impaired glucose tolerance, lower fasting insulin levels, lower counts of enteroendocrine cells, fatty liver, and elevated amounts of hepatic triglycerides, cholesteryl esters, and monounsaturated fatty acids. Lard- and bile acid-fed mice were characterized by shifts in dominant gut bacterial communities, including decreased relative abundances of *Lachnospiraceae* and increased occurrence of *Desulfovibrionaceae* and the species *Clostridium lactatifermentans* and *Flintibacter butyricus*. Metatranscriptomic analysis revealed shifts in microbial functions, including lipid and amino acid metabolism.

**Conclusions:**

Caution is required when interpreting data from diet-induced obesity models due to varying effects of dietary fat source. Detrimental metabolic consequences of a diet enriched with lard and primary bile acids were dependent on microbial colonization of the host and were linked to hepatic lipid rearrangements and to alterations of dominant bacterial communities in the cecum.

**Electronic supplementary material:**

The online version of this article (10.1186/s40168-018-0510-8) contains supplementary material, which is available to authorized users.

## Background

The human intestinal tract harbors trillions of microorganisms referred to as the gut microbiota, which plays an important role in digestion and host metabolism [1] and has been implicated in the development of metabolic diseases, including obesity and type-2 diabetes [2, 3]. However, there is a gap between the increasing number of studies describing changes in ecosystem structure as obtained by sequencing [4] and knowledge about microbial functions and their interactions with diet and host metabolism [5, 6].

Mouse models of diet-induced obesity have been widely used to study microbe-host crosstalk in metabolic diseases. Recent findings pointed at issues related to the robustness of such models, i.e., results are dependent on experimental settings, including animal facilities or diet composition and texture [5, 7–10]. Nonetheless, mouse models are very helpful to test the impact of interventions otherwise not possible in human subjects in terms of, e.g., controlled conditions, invasive sampling, and the ability to address the causal role of changes in the gut microbiome [11–13]. Mouse studies have also helped highlighting the role of single bacterial species in metabolic diseases, such as *Akkermansia muciniphila*, *Christensenella minuta*, *Clostridium ramosum* (recently proposed to be reclassified as *Erysipelatoclostridium ramosum* [14]), *Enterobacter cloacae*, and *Prevotella copri*, including the investigation of underlying molecular mechanisms [15–19].

Several mechanisms by which gut microorganisms can modulate the development of metabolic diseases have been proposed. Interaction with the host via metabolic capacities of the gut microbiota is a particular area of interest, as microbiota members produce myriads of metabolites having many different bioactive properties (e.g., regulation of inflammatory and metabolic responses). Some of the studies aforementioned and several others have demonstrated the importance of short-chain fatty acids (SCFA), branched-chain amino acids, or choline metabolism [18, 20–22]. Bile acid conversion is another important metabolic feature of the gut microbiota with major impact on host metabolism, and the therapeutic potential of intervening with bile acid-dependent pathways has already been exploited in metabolic and inflammatory disorders [23, 24].

Bile acids are cholesterol-derived compounds synthesized in the liver, which facilitate the intestinal absorption of lipids but also influence metabolic and inflammatory signaling pathways, mainly via the farnesoid X receptor (FXR) and G protein-coupled receptor TGR5 [24]. Metabolic disorders have been associated with changes in bile acid composition and concentrations [25, 26]. Moreover, feeding experiments in mice demonstrated that the addition of 0.5% (*w*/*w*) cholic acid (CA) to a high-fat diet (HFD) prevented weight gain and associated comorbidities [27, 28], although underlying interactions with the gut microbiota are unclear. Other studies in rodents demonstrated positive effects of fatty and bile acid conjugates on diet-induced non-alcoholic fatty liver (NAFLD) and hypercholesterolemia [29, 30]. In humans, oral bile acid treatment is common in patients with primary bile acid synthesis deficiency [31], but effects on the gut microbiota are unknown.

Germ-free (GF) and conventional mice markedly differ with respect to bile acid profiles [32]. Intestinal bacteria can transform primary bile acids via deconjugation, dehydroxylation, or dehydrogenation to form the so-called secondary bile acids [33, 34]. Deconjugation reactions are catalyzed by multiple bacterial lineages [35]. In contrast, only a few members of the family *Coriobacteriaceae*, *Clostridiaceae*, *Lachnospiraceae*, or *Ruminococcaceae* are known to produce secondary bile acids, and many of the active strains within these families are not available from public collections for performing downstream experiments to test causal effects [36–38]. Despite this potential of gut microbiota to modulate bile acid bioavailability and the known anti-microbial properties of bile acids [39], only few studies have investigated the impact of primary bile acid supplementation on the gut microbial ecosystem [40, 41].

The source of dietary fat has also been shown to influence host metabolism and microbiota-dependent phenotypes of diet-induced obesity [9, 42, 43]. The response of GF mice to HFD, i.e., their susceptibility to develop diet-induced obesity, depends on the type of high-calorie diet given to the animals, with a particular importance of dietary fat source [8]. Kübeck et al. [9] demonstrated that GF mice fed a HFD based on lard were resistant to diet-induced obesity, whereas those fed palm oil were not due to lower metabolic rate and more efficient fat absorption. The main difference between the two diets was their cholesterol content, with lard-based HFD containing 10 times more. As cholesterol can modulate bile acid and lipid metabolism, these and other authors proposed that dietary cholesterol content drives the response of mice to high-fat diets [9, 44]. Furthermore, dietary fatty acid (FA) composition can modulate body weight gain as well as host metabolism [43, 45]. However, functional implication for the gut microbiota has not yet been described.

The data introduced above indicate that little is known about microbiota-host interactions in response to bile acids and different dietary fat sources. Therefore, the major goal of the present study was to determine the importance of the gut microbiota in regulating the impact of dietary bile acid supplementation on the metabolic status of mice and to test the plasticity of these interactions under conditions of metabolic challenges by using HFDs varying in fat sources (plant or animal). We used both GF and specific pathogen-free (SPF) mice to investigate the impact of microbial colonization. A combination of molecular techniques allowed assessing effects on the host (in particular lipid profiles) and on the composition and functions of intestinal microbial communities.

## Methods

### Mouse experiments

Animal use was approved by the local institution in charge (Regierung von Oberbayern, approval no. 55.2.1.54-2532-156-13). All mice were maintained at the School of Life Sciences Weihenstephan of the Technical University Munich. Male C57BL/6N GF and SPF mice were housed at 22 ± 1 °C and 50–60% relative humidity with a 12-h light/dark cycle and were fed a standard chow diet (V1124-300, Ssniff Spezialdiäten GmbH, Germany). SPF mice were housed in individually ventilated cages whereas cages hosting GF mice were kept in flexible film isolators (North Kent Plastics, UK) ventilated via HEPA-filtered air. To exclude litter and cage effects, mice in each experimental feeding group originated from different litters (three to six litters per group) and were housed in at least three separate cages (one to five mice per cage) (Additional file 1: Figure S1a). Sterility of GF mice was routinely confirmed by culturing and microscopic observation of feces after Gram staining. In addition, 16S rRNA gene-targeted PCR of GF cecal content was performed at the end of the study.

A schematic view of the experimental feeding design is shown in Additional file 1: Figure S1b. Briefly, mice were fed a purified control diet (CD) (Table 1) at 8 weeks of age. After 2 weeks of adaptation to this diet, they were randomly divided into four feeding groups (*n* = 9–12 mice per colonization status per diet) (see all diet compositions in Table 1): (I) CD; (II) CD supplemented with 0.1% (*w*/*w*) cholic acid and 0.1% chenodeoxycholic acid (both ≥ 97% purity; Sigma-Aldrich, Germany) (BA), (III) palm oil-, or (IV) lard-based high-fat diet with 48 kJ% from fat, both supplemented with bile acids as above (P- and LHB, respectively). All diets were purchased from Ssniff Spezialdiäten GmbH, γ-irradiated with 50 kGy, stored at 4 °C after being freshly purchased prior to experiment start, and fed ad libitum to both GF and SPF mice for 8 weeks. At the end of this experimental feeding period, mice were fasted for 6 h. Half of the mice were sacrificed after measurement of fasting blood glucose levels from the tail vein; the other half were used for an oral glucose tolerance test (OGTT) and received therefore 2 g glucose per kg body weight via gavage. Blood glucose levels were measured from the tail vein at 0, 15, 30, 60, and 120 min after gavage and areas under the curve (AUC) of blood glucose levels were calculated for each animal.

**Table 1:** Composition of diets used in the present study

Diet	CD	BA	PHB	LHB
Product number	S5745-E902	S5745-E905	S5745-E915	S5745-E935
Energy [MJ/kg]	15.3	15.3	19.7	19.7
Fat [kJ%]	13	13	48	48
Protein [kJ%]	23	23	18	18
Carbohydrates [kJ%]	64	64	34	34
Casein [%]	24.0	24.0	24.0	24.0
Corn starch [%]	47.8	47.6	27.8	27.8
Maltodextrin [%]	5.6	5.6	5.6	5.6
Saccharose [%]	5.0	5.0	5.0	5.0
Cellulose[%]	5.0	5.0	5.0	5.0
L-Cystin [%]	0.2	0.2	0.2	0.2
Vitamins [%]	1.2	1.2	1.2	1.2
Minerals/trace elements [%]	6.0	6.0	6.0	6.0
Cholin-Cl [%]	0.2	0.2	0.2	0.2
Soy oil [%]	5.0	5.0	5.0	5.0
Palm oil [%]	–	–	20.0	–
Pork lard [%]	–	–	–	20.0
Cholic acid [%] ^a^	–	0.1	0.1	0.1
Chenodeoxycholic acid [%] ^b^	–	0.1	0.1	0.1
Fatty acid composition [%]				
C12:0	0.01	0.01	0.01	0.05
C14:0	0.02	0.02	0.21	0.29
C16:0	0.58	0.58	9.18	5.37
C18:0	0.18	0.18	1.11	2.88
C20:0	0.02	0.02	0.10	0.08
C16:1	0.01	0.01	0.05	0.60
C18:1	1.29	1.29	9.19	9.64
C18:2	2.65	2.65	4.67	4.55
C18:3	0.29	0.29	0.35	0.49

All diets were purchased from Ssniff Spezialdiäten GmbH; ^a^Sigma-Aldrich, cat. no. C1129; ^b^Sigma-Aldrich, cat. no. C9377; *CD* control diet, *BA* control diet supplemented with 0.2% (*w*/*w*) primary bile acids, *PHB* palm oil-based HFD supplemented with bile acids, *LHB* lard-based HFD supplemented with primary bile acids, *C* carbon

### Sampling

All mice were sacrificed with carbon dioxide. Systemic EDTA blood was collected from the vena cava and centrifuged (3000×*g*, 4 °C, 10 min). Plasma was aliquoted and snap-frozen in liquid nitrogen. Organs were dissected, their weight was recorded, and they were either directly snap-frozen in liquid nitrogen or fixed in 4% formalin for 48 h. Epididymal, mesenterial, and inguinal white adipose tissues (WAT) were collected and weighed, and total WAT mass, i.e., the sum of all three tissues referred to as “WAT mass” hereon, was calculated. Intestinal content or tissue from different gut regions was collected into sterile tubes and immediately snap-frozen in liquid nitrogen. Frozen samples were stored at − 80 °C until analysis.

### Serum insulin and leptin measurement

Systemic plasma insulin and leptin concentrations were determined using a Luminex 100 IS system (Luminex Corporation) with a Milliplex MAP mouse serum adipokine panel kit (Merck Millipore), as described previously [46].

### Liver histopathology

Formalin-fixed paraffin-embedded liver samples were cut into 5-μm-thick sections using a Leica rotary microtome RM2255, mounted on SuperFrost® microscope slides (Thermo Fisher Scientific) and dried overnight. Sections were then heat-treated (15 min, 60 °C) to melt paraffin and trichromatically stained with hematoxylin, eosin, and saffron dyes with a multistainer station (Varistain™, Thermo Fisher Scientific Inc., Germany). Once covered with a glass cover slip, virtual slides were made by using the Pannoramic Scan 150 (3DHISTECH Ltd., Hungary) and examined in a blinded manner using a semi-quantitative scoring system. Briefly, steatosis (0–3 points), lobular inflammation (0–3), and ballooning (0–2) of hepatocytes were evaluated. Points were summed up to obtain a total fatty liver activity score, which ranged from 0 (no pathology) to 8 (severe disease) [47].

### Immunohistochemical staining for glucagon-like peptide 1 and chromogranin A

Sections (5 μm) of paraffin-embedded tissue from the proximal colon were used. At least three non-consecutive sections were stained from each mouse. After deparaffinization, antigen retrieval was performed by boiling in citrate buffer. Glucagon-like peptide (GLP) 1 and chromogranin A (ChgA) antibodies (Santa Cruz Biotechnology) were diluted 1:75 and applied overnight at 4 °C. The secondary antibody (mouse anti-goat, dianova) was diluted 1:300 and slides were incubated for 1 h at room temperature. For development, 3,3′-diaminobenzidine (DAB) or enhanced DAB (Sigma Aldrich) were applied for ChgA and GLP-1 stainings, respectively. Slides were subsequently counterstained with hematoxylin and mounted with xylol-based mounting medium (Roti®-Histokitt). GLP1-positive (GLP1+) and ChgA-positive (ChgA+) cells were quantified using a PreciPoint M8 microscope.

### qPCR analysis of liver mRNA expression

Total RNA was extracted from liver samples using the RNeasy Mini kit (Qiagen). RIN (RNA integrity number) values were assessed with an Agilent 2100 Bioanalyzer using the RNA 6000 Nano Kit. Total RNA (10 μg) was reverse transcribed using random primers and a High-Capacity Complementary DNA Reverse Transcription Kit (Applied Biosystems). Pre-amplification of cDNA was then performed using the TaqMan® PreAmp Master Mix (Applied Biosystems). The final cDNA samples were stored at − 20 °C until RT-qPCR was performed using the TaqMan® Gene Expression Technology (Applied Biosystems). Probes were as follows: Mm00432403_m1 (*Cd36*), Mm00440939_m1 (*Ppar-α*), Mm00440940_m1 (*Ppar-γ*), Mm01304257_m1 (*Acaca*), and Mm02342723_m1 (*Mlxipl*). DNA was amplified using the StepOne Plus Real-Time PCR system (Applied Biosystems). Data were recorded by the manufacturer’s software and the RQ Manager Analysis Software (Applied Biosystems) was used to determine Ct values. GAPDH was identified as the least variable housekeeping gene and was chosen to normalize data in this study. Relative quantification of gene expression was calculated by means of ddCt values (2^−[(Ct^_target gene_ ^− Ct^_GAPDH_^)treated − (Ct^_target gene_ ^− Ct^_GAPDH_^)untreated]^).

### Hepatic triglyceride content

Portions of frozen liver were homogenized in chloroform-methanol (2:1) to extract total lipids as previously described [48]. The organic extract was dried and reconstituted in isopropanol. Triglycerides were quantified using a serum triglyceride determination kit (TR0100, Sigma-Aldrich, Germany) and expressed as milligram per gram liver.

### Fatty acid analysis

Analysis of total fatty acids (FA) was performed as described previously [49]. Briefly, fatty acid methyl esters (FAMEs) were generated with acetyl chloride and methanol overnight at room temperature and extracted with hexane. Total FA analysis was carried out using a Shimadzu 2010 GC-MS system (Shimadzu Deutschland GmbH, Germany). FAMEs were separated using a BPX70 column (10-m length, 0.10-mm diameter, 0.20-μm film thickness; SGE Analytical Science Europe Ltd., UK) using helium as carrier gas. The initial oven temperature was 50 °C, which was programmed to increase with 40 °C per min to 155 °C, with 6 °C per min to 210 °C, and with 15 °C per min to finally reach 250 °C. FA species and their positional and cis/trans isomers were characterized in scan mode and quantified by single-ion monitoring mode detecting the specific fragments of saturated and unsaturated FAs (saturated: *m*/*z* 74; monounsaturated: *m*/*z* 55; diunsaturated: *m*/*z* 67; polyunsaturated: *m*/*z* 79). Non-naturally occurring *iso*-C21:0 was used as an internal standard.

### Glycerophospholipid and cholesterol analysis

Lipids were extracted according to a procedure described by Bligh and Dyer in the presence of non-naturally occurring lipid species as internal standards [50]. Lipids were quantified by electrospray ionization tandem mass spectrometry (ESI-MS/MS) in positive ion mode as described previously [51]. In brief, samples were analyzed by direct flow injection using a HTS PAL autosampler, an Agilent 1100 binary pump (Germany), and triple quadrupole mass spectrometer (Quattro Ultima, Micromass, Germany). A precursor ion scan of *m*/*z* 184 specific for phosphocholine containing lipids was used for phosphatidylcholine (PC), sphingomyelin (SM), and lysophosphatidylcholine (LPC) [52]. The following neutral losses were applied: phosphatidylethanolamine (PE) 141, phosphatidylserine (PS) 185, phosphatidylglycerol (PG) 189, and phosphatidylinositol (PI) 277 [53, 54]. PE-based plasmalogens (PEP) were analyzed according to the principles described by Zemski-Berry [55]. Sphingosine-based ceramides (Cer) were analyzed using a fragment ion of *m*/*z* 264 [56]. Free cholesterol (FC) and cholesteryl ester (CE) were quantified using a fragment ion of *m/z* 369 after selective derivatization of FC using acetyl chloride [57]. Correction of isotopic overlap of lipid species and data analysis by Excel Macros was performed for all lipid classes. Quantification was performed by standard addition calibration to cell homogenates using a number of naturally occurring lipid species for each lipid class. Lipid species were annotated according to the recently published proposal for shorthand notation of lipid structures that are derived from mass spectrometry [58]. Glycerophospholipid species annotation was based on the assumption of even-numbered carbon chains only.

### Bile acid measurement

Bile acids were quantified in blood according to our recently described method [38]. Briefly, 50 μl EDTA-plasma was mixed with 125 μl methanol and 25 μl internal standard (IS) working solution (100 μM d4-CA, 100 μM d4-GCDCA, and 1000 μM d7-Chol), vortexed, and shaken continuously for 10 min. After centrifugation (12,000×*g*, 4 °C, 10 min), 100 μl supernatant were transferred into a new glass vial, evaporated to dryness under a gentle stream of nitrogen, and redissolved in 50 μl methanol. The analysis of bile acids and cholesterol was performed on an Agilent 1260 Infinity Quaternary LC System (Agilent Technologies Deutschland GmbH & Co. KG, Germany) coupled to a triple quadrupole API 4000 QTRAP® MS (AB Sciex Germany GmbH) equipped with a turbo ion spray source, operating either in positive or negative ion mode. A Kinetex® C18 reversed phase column equipped with a Kinetex® C18 security guard column (Phenomenex Inc., Germany) was used for separation of the analytes (constant flow rate of 200 μl/min).

### Quantitation of short-chain fatty acids (SCFAs)

SCFA measurement was performed by LC-MS/MS after 3-nitrophenylhydrazine derivatization using a recently reported method with some modifications [59]. Frozen fecal samples (5–20 mg) were precisely weighed, suspended in 1 ml of an internal standard solution containing propionic acid-d5, ^13^C_2_-acetate, and ^13^C_4_-butyrate in acetonitrile/water (1 + 1, *v*/*v*, 1 ml), and homogenized by vortexing after addition of glass beads (10 beads, diameter 2 mm). After equilibration (30 min) on an orbital shaker, samples were centrifuged (12,000 rpm, 4 °C), and supernatants (40 μl) were placed into autosampler vials, mixed with 20 μl of 3-nitrophenylhydrazine hydrochloride (200 mmol/l) in acetonitrile/water (1/1, *v*/*v*) and 20 μl of N-(3-dimethylaminopropyl)-N′-ethylcarbodiimide hydrochloride (120 mmol/L) in acetonitrile/water (1/1, *v*/*v*) containing 6% pyridine. After 30 min at 40 °C, samples were diluted with acetonitrile/water (1/9, *v*/*v*; 200 μl) and aliquots (1 μl) were used for UHPLC-MS/MS analysis.

A Nexera X2 UHPLC system (Shimadzu, Duisburg, Germany), consisting of two LC pumps LC30AD, a DGU-20 degasser, a SIL-30AC autosampler, a CTO-30A column oven, and a CBM-20A system controller, was hyphenated with a QTRAP 6500 LC-MS/MS system (Sciex, Darmstadt, Germany). Chromatographic separation was performed on a Kinetex C18 column (100 × 2.1 mm, 1.7 μm, 100 Ǻ, Phenomenex, Aschaffenburg, Germany) using water/formic acid (100/0.1, *v*/*v*) as solvent A and acetonitrile/formic acid (100/0.1, *v*/*v*) as solvent B at a flow rate of 0.35 ml/min and a column temperature of 40 °C. Starting with initial conditions of 17% B for 2 min, the content of B in the mobile phase was increased to 60% within 9 min, followed by an immediate switch to 100% B (held for 1 min), and re-equilibration at starting conditions for 3 min.

The mass spectrometer was operated in the negative electrospray ionization and low mass mode, and the ion spray voltage was set at − 4500 V. Nitrogen served as nebulizer gas (55 psi), turbo gas (500 °C) for solvent drying (65 psi), curtain gas (35 psi), and collision gas (1.9 × 10^− 5^ Torr). The MS/MS parameters, declustering potential, entrance potential, collision cell entrance potential, collision energy, and cell exit potential were tuned for each individual compound after derivatization by flow injection (10 μl/min), detecting the fragmentation of the [M-H]-molecular ions into specific product ions after collision with nitrogen (4.5 × 10^− 5^ Torr). Mass spectrometric data were analyzed using Analyst software 1.6.2 (Sciex). Target analytes were detected based on scheduled MRM mode using the following mass transitions: 3-NPH-acetate (*m*/*z* 193.9 → 136.8), 3-NPH-propanoate (*m*/*z* 207.9 → 136.8), 3-NPH-butyrate (*m*/*z* 221.9 → 136.9), 3-NPH-isobutyrate (*m*/*z* 222.0 → 136.9), 3-NPH-valerate (*m*/*z* 236.0 → 136.8), 3-NPH-isovalerate (*m*/*z* 236.0 → 137.0), 3-NPH-2-methylbutyrate (*m*/*z* 236.0 → 136.8), 3-NPH-hexanoate (*m*/*z* 250.0 → 136.7), and 3-NPH-4-methylvalerate (*m*/*z* 250.0 → 136.9). While acetate and propanoate were quantified using their isotopologues 3-NPH-^13^C_2_-acetate (*m*/*z* 196.0 → 136.9) and 3-NPH-d5-propionate (*m*/*z* 213.0 → 136.9), the remaining SCFAs were determined using 3-NPH-^13^C_4_-butyrate (*m*/*z* 226.0 → 137.0) as internal standard. After UHPLC-MS/MS analysis, calibration curves (0.0001–1.6 mg/l; eight-point calibration) were prepared by plotting peak area ratios of analyte to internal standard against concentration ratios of each analyte to the internal standard using linear regression (*R*_2_ > 0.997). For each sample, data were calculated as the means of triplicate analysis.

### Bacterial cultivation

For determination of viable bacterial cell counts, sample processing and incubation were carried out under anaerobic conditions (N2/H2, 90:10) in a Whitley H85 workstation. Materials were brought into the workstation at least 24 h prior to experiments. Fresh cecal content was weighed and diluted 1:10 with filter-sterilized phosphate-buffered saline (PBS) containing 0.02% (*w*/*v*) peptone and 0.05% L-cystein. After preparation of serial 1:10-dilution series (one per sample), 10 μl of each dilution were plated onto Wilkins-Chalgren-Anaerobe (WCA) agar (Oxoid) supplemented with filter-sterilized 0.02% dithiothreitol (DTT) and 0.05% L-cystein. Plates were incubated at 37 °C for 1 week (SPF mice) or 2 weeks (GF mice). Colony-forming units (CFUs) were enumerated and expressed per gram of cecal content (wet weight).

### DNA isolation

Metagenomic DNA was obtained from cecal content of fasted SPF mice after mechanical lysis followed by purification according to a published protocol [60] modified as follows: cecal content in 600 μl stool DNA stabilizer (Stratec Biomedical AG) was transferred into a 2-ml screw-cap tube containing 500 mg zirconia/silica beads (0.1 mm; BioSpec Products), 250 μl 4 M Guanidinethiocyanate (Sigma-Aldrich, Germany), and 500 μl 5% N-lauroylsarcosine (Sigma-Aldrich, Germany). Samples were mixed and incubated for 60 min at 70 °C with constant shaking, and bacterial cells were disrupted by mechanical lysis using a FastPrep®-24 (three times, 40 s, 6.5 m/sec) (MP Biomedicals) fitted with a cooling adaptor. After addition of 15 mg polyvinylpolypyrrolidone (PVPP, Sigma-Aldrich, Germany), the suspension was vortexed and centrifuged (3 min, 15,000×*g*, 4 °C). The supernatant (500 μl) was transferred into a new Eppendorf tube, mixed with 5 μl RNase (VWR International, stock concentration 10 mg/ml) and incubated for 20 min at 37 °C with constant shaking. Genomic DNA was purified using NucleoSpin® gDNA columns (Macherey Nagel GmbH & Co. KG, Germany) following the manufacturer’s instructions. DNA quantity and quality were measured with a NanoDrop® instrument (Thermo Fisher Scientific Inc., Germany).

### 16S rRNA gene-targeted PCR

To test the sterility of GF mice, 16S rRNA genes were amplified using primer 27F (5′-agagtttgatcctggctcag) and 1492R (5′-ggttaccttgttacgactt) [61]. For each sample, the PCR mixture contained 25 ng DNA, 20 μl 2× DreamTaq green PCR mastermix (Thermo Fisher Scientific Inc., Germany), and 1 μl of each primer stock solution (20 μM). PCR conditions were 3 min at 95 °C followed by 25 cycles of 95 °C for 30 s, 55 °C for 30 s, 72 °C for 90 s, and a final extension at 72 °C for 5 min. PCR products were separated by electrophoresis in 1% agarose gels and visualized using the GeneFlash system (Syngene International Ltd.).

### High-throughput 16S rRNA gene amplicon analysis

Libraries were constructed in a semi-automated manner using a Biomek-4000 pipetting robot (Beckmann Coulter Biomedical GmbH). The V3/V4 region of 16S rRNA genes was amplified (25 cycles) from 24 ng of metagenomic DNA using primer 341F and 785R in a two-step procedure to limit amplification bias [62, 63]. Libraries were double-barcoded (8-nt index on each of the forward and reverse 2nd-step primer) [64, 65]. Amplicons were purified using the AMPure XP system (Beckmann Coulter Biomedical GmbH), pooled in an equimolar amount with addition of 25% (*v*/*v*) PhiX library, and sequenced in paired-end modus (PE275) using a MiSeq system (Illumina).

Data were analyzed as described in detail previously [66]. Raw sequence reads were processed using IMNGS (www.imngs.org) [67], an in-house-developed pipeline based on UPARSE [68]. Parameters were as follows: barcode mismatches, 2; expected error, 3; Phred quality threshold, ≥ 3; trimming score, 3; trimming length, 10 nt; min. sequence length, 300 nt; max. sequence length, 600 nt (see IMNGS website for further information). Operational taxonomic units (OTUs) were clustered at 97% sequence similarity and only those occurring at a relative abundance ≥ 0.25% total reads in at least one sample were further analyzed. For each OTU, the final taxonomy was assigned using the most detailed classification among SILVA [69] and RDP [70].

### Metatranscriptomics

Total RNA was extracted from frozen cecal contents as follows: approx. 50 mg content was mixed with 300 μl RLT buffer supplemented with B-mercaptoethanol (10 μl/ml, Sigma-Aldrich, cat. no. M3148) and 1 ml Trizol (Invitrogen, cat. no. 15596-18) and vortexed for 15 s. RNase- and DNase-free glass beads (600 mg, Sigma-Aldrich, cat. no. G4649-100G) were added prior to cell disruption using a FastPrep®-24 (40 s then 20 s at 6.5 m/sec) (MP Biomedicals). After 5 min at room temperature and centrifugation (1 min, 12,000×*g*, 4 °C), supernatants were transferred into a tube containing 300 μl chloroform (VWR, cat. no. 22711290), vortexed, incubated 3 min at room temperature, and centrifuged (15 min, 12,000×*g*, 4 °C). The aqueous phase was carefully collected and transferred into a new tube containing 1 ml of freshly prepared 70% ethanol solution. Tubes were inverted five times and the mixture was loaded onto a RNeasy spin column (RNeasy mini kit, Qiagen, cat. no. 74104). RNA extraction was completed as described by the manufacturer including on-column DNA digestion using the RNase-free DNAse set (Qiagen, cat. no. 79254). Total RNA was depleted from rRNA using the Ribo-Zero™ Bacteria Kit (Illumina, cat. no. MRZB12424) as recommended by the manufacturer. rRNA-depleted RNA was purified using the RNeasy MinElute CleanUp Kit (Qiagen, cat.no. 74204). cDNA synthesis and library preparation were performed using the ScriptSeq™v2 RNA-Seq Library Preparation Kit (epicenter, cat. no. SSV21106/SSV21124). cDNA was purified using the MinElute PCR Purification Kit (Qiagen, cat. no. 28004). Libraries were multiplexed and sequenced on a HiSeq2500 sequencer (Illumina) with Rapid v2 chemistry and the 2×150 bp paired-end read module. Raw reads were checked for quality scores (*Q* ≥ 25) and length (*L* ≥ 100 bp) using sickle (https://github.com/najoshi/sickle). Residual ribosomal reads were removed using SortMeRNA [71]. mRNA reads were mapped onto an in-house-implemented mouse metagenome catalog based on Xiao et al. [72] and containing 4.5 million genes using bwa [73]. Mapping results of the metatranscriptomic dataset were analyzed using DESeq2 [74].

### Statistics

Unless otherwise stated, data are presented as mean ± SD. Statistics were performed in R or using Prism version 7.00 (GraphPad). The latter software was also used for generating graphs. The following statistical tests were used: (I) Effects of feeding and colonization groups were compared using two-way ANOVA followed by pairwise testing (Holm-Sidak; *p < 0.05; **p < 0.01; ***p < 0.001), (II) Effects of diets within one colonization group or of colonization status for a given diet were compared using one-way ANOVA followed by pairwise testing (Holm-Sidak; ^**#**^*p* < 0.05; ^**##**^*p* < 0.01; ^**###**^*p* < 0.001). Regression analysis was performed by ANCOVA in Microsoft Office Excel 2016 with pairwise comparison. Statistical analysis of microbiota data was performed in Rhea [75]. EzTaxon [76] was used for the identification of OTUs showing significant differences (*p* < 0.05) in relative abundances between feeding groups.

## Results

### Metabolic state depends on dietary fat source and the presence of intestinal microbes

We first characterized the metabolic status of mice following dietary bile acid supplementation with or without addition of fat (derived from plant or animal) in the presence (SPF) or absence (GF) of gut commensals.

**Fig. 1:**
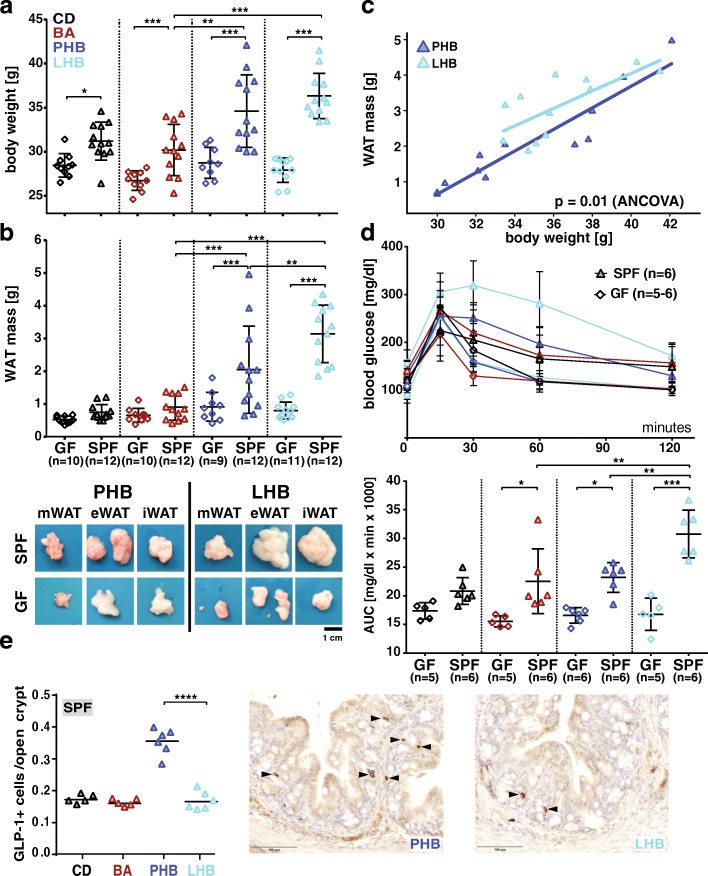
Impact of experimental feedings and microbial colonization on mouse metabolism. **a** Final body weight (at the age of 18 weeks after 8 weeks of feeding). **b** WAT mass for all groups and representative pictures of respective fat depots collected from SPF mice fed the palm oil- or lard-based diets. **c** Corresponding regression analysis of WAT mass and body weight. **d** Blood glucose concentrations during OGTT with corresponding areas under the curve. Color code for diets: CD, black; BA, red; PHB, dark blue; LHB, cyan blue. Symbols for colonization status: GF, diamonds; SPF, filled triangles. All mice used in the experiments are shown (group size varied as indicated below the *x*-axis). See the “Methods” section for description of statistical analyses. **e** Quantification of glucagon-like peptide (GLP) 1-positive cells in colonic tissue sections of SPF mice from the different feeding groups. At least three non-consecutive sections were stained from each mouse and quantified. Symbols represent average values from individual mice. Representative pictures of immunohistochemical staining acquired with a confocal microscope are shown (for the sake of space and appropriate size of images, picture for CD and BA are not shown but are equivalent to LHB group). Arrows indicate cells positive for GLP1. The black bars indicate 100 μm. ***p < 0.01, one-way ANOVA followed by the Tukey test (performed using Graph Pad Prism)

SPF mice were significantly heavier than age-matched GF mice in all diet groups at the end of the feeding period (18 weeks of age) (Fig. 1a). Bile acid supplementation did not influence body weight, whereas both HFDs increased body weight when combined with BA for 8 weeks. This HFD-induced body weight gain was observed only in SPF, not in GF mice (Fig. 1a and Additional file 2: Figure S2a). Interestingly, SPF mice fed lard (LHB) were characterized by a higher increase in WAT mass compared to palm oil (PHB) (3.2 ± 0.9 vs. 2.0 ± 1.3 g; *p* = 0.0014) (Fig. 1b). This observation was confirmed by regression and ANCOVA analyses (Fig. 1c). To assess metabolic consequences of this difference in fat mass, we performed an OGTT that revealed a significant effect of dietary fat: lard-fed SPF mice were characterized by impaired glucose tolerance when compared to the palm oil group (Fig. 1d). Similar to body weight gain, glucose tolerance was not affected by the different diets in GF mice. Regression and ANCOVA analyses of fasting blood insulin and leptin levels indicated lower concentrations of insulin in LHB- vs. PHB-fed SPF mice (*p* = 0.0009) (Additional file 2: Figure S2b). There was no difference for leptin (*p* = 0.523).

Gut-derived incretin hormones produced by enteroendocrine cells (EEC) influence glucose tolerance and insulin secretion. Because GLP-1 is produced by a subset of enteroendocrine cells (EEC) located in the epithelium of the lower gastrointestinal tract, we quantified numbers of cells positive for GLP-1 and the EEC-marker chromogranin A (ChgA) in colonic sections from the different feeding groups. Palm oil feeding combined with bile acids was associated with an increase in both GLP1-positive cells (Fig. 1e) and total EEC numbers (Additional file 2: Figure S2c), whereas lard showed no alterations compared to the CD and BA groups.

Altogether, the data aforementioned indicate that lard in the diet had a detrimental impact on host metabolism when combined with bile acids, but only in the presence of endogenous gut microbes.

### Dietary lard alters host lipid profiles

**Fig. 2:**
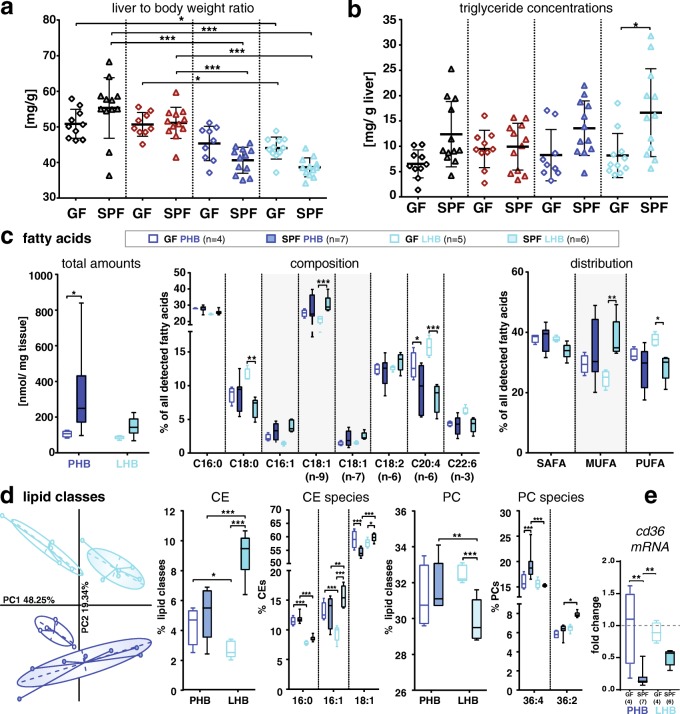
Modulation of hepatic lipid profiles. **a** Liver to body weight ratio. **b** Triglyceride content. **c** Amounts, composition, and distribution of total fatty acids measured in fasted mice (*n* = 4–7 as indicated in the figure). **d** Alterations in lipid classes and species. Only lipids representing > 1% total amounts were considered for statistical analysis. Color code is as in Fig. 1. SAFA saturated fatty acids, MUFA monounsaturated fatty acids, PUFA polyunsaturated fatty acids, unsat unsaturated fatty acids, PC phosphatidylcholine, CE cholesteryl esters. See the “Methods” section for description of mass spectrometric measurements and for statistical analyses. **e** mRNA relative expression of *Cd36* in the liver of mice. GAPDH was used as housekeeping gene for the normalization. GF mice fed the PHB diet were used as reference group. In all figure panels, stars indicate statistical significance as follows: *p < 0.05; **p < 0.01; ***p < 0.001 (two-way ANOVA followed by Holm-Sidak)

We then looked more specifically at the liver as the central organ for lipid, bile acid, and cholesterol metabolism. The combination of HFD and BA feeding for 8 weeks reduced liver to body weight ratios, independent of dietary fat source (Fig. 2a). This decrease was not due solely to increased body weight but indeed to lower liver weight, as shown by regression analysis (Additional file 3: Figure S3a). Liver histopathology revealed that HFD-induced fatty liver activity scores were higher in SPF vs. GF mice, which was significant only for the lard diet (Additional file 3: Figure S3b). This was due to more severe steatosis but not to inflammation and ballooning (data not shown). In line with the changes observed in body weight between GF and SPF mice, the colonization status of mice influenced hepatic triglyceride concentrations, which were higher in SPF vs. GF mice fed the CD, PHB, and LHB diets, yet significance was reached only for the lard-fed group (Fig. 2b).

To pinpoint specific changes associated with the lard-induced metabolic effects described in Fig. 1, we determined hepatic lipid profiles in GF and SPF mice fed the PHB vs. LHB diets. Total amounts of fatty acids were 3.3- (PHB) and 1.7-fold (LHB) higher in the liver of SPF vs. GF mice, without significant changes between the two HFDs (Fig. 2c). With respect to fatty acid composition, the amplitude of colonization-induced changes (SPF vs. GF) was higher in the lard-fed group. SPF LHB mice showed significantly higher proportions of monounsaturated fatty acids (MUFA), in particular oleic acid (FA 18:1 n-9), and lower proportions of poly-unsaturated fatty acids (PUFA), in particular arachidonic acid (FA 20:4 n-6) (Fig. 2c). Since the major fraction of hepatic fatty acids are esterified to cell membrane lipids and sterols, we next analyzed glycerophospholipid, sphingolipid, and cholesteryl ester (CE) species. Principal component analysis (PCA) highlighted colonization- and diet-specific profiles (Fig. 2d). Dietary fat source significantly affected total phosphatidylcholine (PC) and CE levels in SPF mice. Total CE fractions were 1.8-fold higher in LHB mice (including higher proportions of CE 16:1 and CE 18:1), while PC proportions were decreased, with higher representation of PC 36:2 vs. 36:4.

As we observed significant changes in hepatic lipid profiles between animals fed the two HFDs, we quantified the expression of genes involved in lipid transport and metabolism in liver samples. The expression of *Cd36*, encoding a scavenger receptor involved in long-chain fatty acid transport, was decreased approx. ten- and twofold in SPF mice fed the PHB and LHB, respectively, when compared to corresponding GF mice, without statistically significant difference between the two HFDs (Fig. 2e). There was also no significant colonization- or diet-induced differences in expression of the other genes measured (*Ppar-α*, *Ppar-γ*, *Acaca*, *Mlxipl*). Lipid analysis also included the quantification of cholesterol and bile acids in the blood (Table 2) [38]. The sole fat source-dependent difference in SPF mice was significantly increased systemic concentrations of tauro-chenodeoxycholic acid (TCDCA) in lard- vs. palm oil-fed animals (21.5 ± 12.6 nM vs. 7.1 ± 8.4 nM, *p* = 0.0415, *t* test). Cholesterol levels were neither affected by the colonization status nor by the diet.

**Table 2:** Bile acid and cholesterol concentrations in systemic plasma of fasted mice

Colonization status	GF		SPF	
Diet	PHB	LHB	PHB	LHB
T-α-MCA [nM]	107 ± 60	163 ± 128	31 ± 11	53 ± 31
T-β-MCA [nM]	*358 ± 125* ^a^	*780 ± 617*	*36 ± 10* ^c^	*34 ± 17* ^d^
TCA [nM]	186 ± 116	*236 ± 173*	39 ± 54	*35 ± 23* ^d^
TCDCA [nM]	23 ± 28	43 ± 51	*7.1 ± 8.4*	*22 ± 13* ^b^
TDCA [nM]	BQ	BQ	53 ± 19	83 ± 32
β-MCA [nM]	29 ± 26	134 ± 225	31 ± 40	21 ± 37
12-DHCA [nM]	BQ	BQ	2.2 ± 4.0	1.6 ± 3.8
CA [nM]	3.8 ± 6.5	7.6 ± 10.8	45 ± 39	37 ± 25
TLCA [nM]	BQ	BQ	BQ	BQ
DCA [nM]	BQ	BQ	103 ± 32	142 ± 55
SUM of all bile acids [nM]	706 ± 348	*1363 ± 1179*	347 ± 95	*429 ± 151* ^d^
Primary [nM]	706 ± 348	*1363 ± 1179*	189 ± 87	*202 ± 103* ^d^
Secondary [nM]	BQ	BQ	159 ± 48	227 ± 85
Tauro-conjugated [nM]	673 ± 326	*1221 ± 963*	166 ± 59	*228 ± 61* ^d^
Unconjugated [nM]	33 ± 28	142 ± 220	181 ± 83	201 ± 104
Cholesterol [μM]	628 ± 241	539 ± 234	775 ± 242	790 ± 235

Data are mean ± SD. Diets are as in Table 1. Italicized data indicate differences between groups. Superscript letters indicate statistical significance (*p* < 0.05, *n* = 4–6; two-way ANOVA with Holm-Sidak for multiple comparison; *t* test for LHB vs. PHB comparisons within colonization groups) as follows: ^a^P- vs. LHB among GF mice; ^b^P- vs. LHB among SPF mice; ^c^GF vs. SPF for PHB diet; ^d^GF vs. SPF for LHB diet. *CA* cholic acid, *CDCA* chenodeoxycholic acid, *DCA* deoxycholic acid, *DHCA* dihydroxycholic acid, *LCA* lithocholic acid, *MCA* muricholic acid, *T* tauro-conjugated, *BQ* below quantification limit [38]

In summary, alterations of the mouse metabolic status associated with dietary lard in combination with bile acids were accompanied by significant changes in lipid profiles. The observation that diet effects were absent in GF mice implied that microbial colonization is at least partly responsible for the changes observed, which prompted us to analyze gut microbiota structure and functions.

### Dietary fat and bile acid supplementation modulates the mouse cecal microbiota

#### Microbiota structure

One known major difference between GF and SPF mice is reduced cecum weight under SPF conditions, which was also observed in the present study (Additional file 4: Figure S4A). In contrast, the effect of bile acid supplementation on cecum weight had not been investigated so far. BA feeding significantly reduced cecum weight in both GF and SPF mice, and this decrease was accentuated by HFDs. Anaerobic cultivation of cecal contents confirmed the germ-free status of GF mice (Additional file 4: Figure S4B), which was also supported by negative 16S rRNA gene-targeted PCRs (Additional file 4: Figure S4C). Cultivation also showed that the different diets did not significantly alter viable bacterial counts in SPF mice (Additional file 4: Figure S4B).

High-throughput sequencing of 16S rRNA gene amplicon libraries was performed to obtain first insights into diet-induced shifts in gut bacterial profiles. We analyzed samples from fasted SPF mice only (*n* = 6–7) to exclude confounding effects of oral glucose in the group subjected to OGTT. A total of 475,710 quality- and chimera-checked sequences (19,028 ± 2768 per sample) representing a total of 153 operational taxonomic units (OTUs) (125 ± 6 per sample) were obtained and further analyzed (Additional file 5: Table S1).

**Fig. 3:**
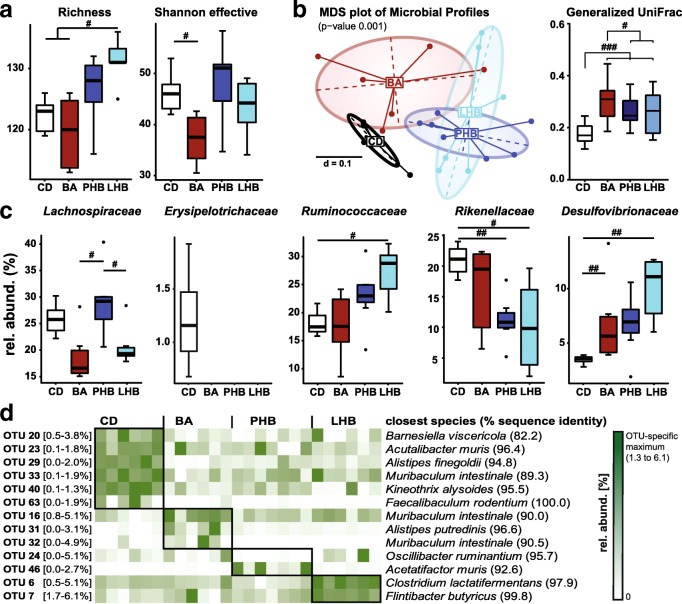
Diet-induced alteration of cecal microbiota profiles. **a** Alpha-diversity shown as richness and Shannon effective counts. **b** Beta-diversity analysis via multidimensional scaling analysis of generalized UniFrac distances. The *p* value was obtained by PERMANOVA for testing the significance of separation between sample groups. **c** Boxplots of significantly altered taxonomic groups at the family level. *Erysipelotrichaceae* were detected in four of six CD-fed mice. **d** Relative abundances of dietary group-specific OTUs shown as a heat map. OTU sequences (ca. 450 bp of the V3/V4 region) were classified using EzTaxon. The range of relative abundances of each OTU is given in square brackets next to the corresponding OTU identification number. Statistics were performed and original graphs were generated in the R programming environment using Rhea [67]: *p < 0.05; **p < 0.01; ***p < 0.001. Number of mice: CD, 6; BA, 6; PHB, 7; LHB, 6

LHB feeding was associated with increased richness (ca. 10 molecular species) when compared to both BA and CD, but not significantly to PHB (Fig. 3a). BA did not affect richness, yet Shannon effective counts were decreased significantly, which suggests shifts in the evenness of dominant species distribution. Beta-diversity analysis revealed a significant clustering of samples according to diet (Fig. 3b). In particular, all experimental diets increased inter-individual differences in the phylogenetic makeup of cecal microbiota (i.e., within group heterogeneity) when compared to the control diet, suggesting less stable states of the ecosystem. Diet-induced shifts in microbiota composition were clearly visible at the family level: all dietary interventions (BA, PHB, and LHB) were associated with increased proportions of *Desulfovibrionaceae*, whereas *Erysipelotrichaceae* were not detected in these mice (Fig. 3c). The relative abundance of *Lachnospiraceae* was discriminative between the palm- and lard-based intervention (ca. 15% decrease in the latter group) and that of *Ruminococcaceae* was higher in LHB vs. CD. Both PHB and LHB showed lower relative abundances of *Rikenellaceae*.

A deeper look at the level of single molecular species showed that the four dietary interventions were characterized by the presence of specific OTUs (Fig. 3d). Within the family *Erysipelotrichaceae*, *Faecalibaculum rodentium* was specific to the control diet, while BA-fed mice exhibited higher proportions of OTUs most closely related to *Alistipes* and *Muribaculum* species. Significant differences were also observed between the two HFDs: palm oil feeding increased the relative abundance of one OTU with closest match to *Acetatifactor muris*, whereas *Oscillibacter ruminantium* was not detectable in this group. Mice fed the lard-based diet were characterized by increased relative abundances of *Clostridium lactatifermentans* and *Flintibacter butyricus.*

Taken together, bile acids and dietary fat source affected cecal microbiota structure. Hence, we further investigated microbial functions. Measurement of short-chain fatty acid (SCFA) in colonic content of SPF mice indicated higher concentrations of acetate in PHB mice, but results did not reach significance and the colonic concentrations of all other SCFA were also not affected (Additional file 6: Figure S5). In order to obtain a comprehensive view of microbial functions, cecal contents were further analyzed using metatranscriptomics.

#### Microbiota functions

**Fig. 4:**
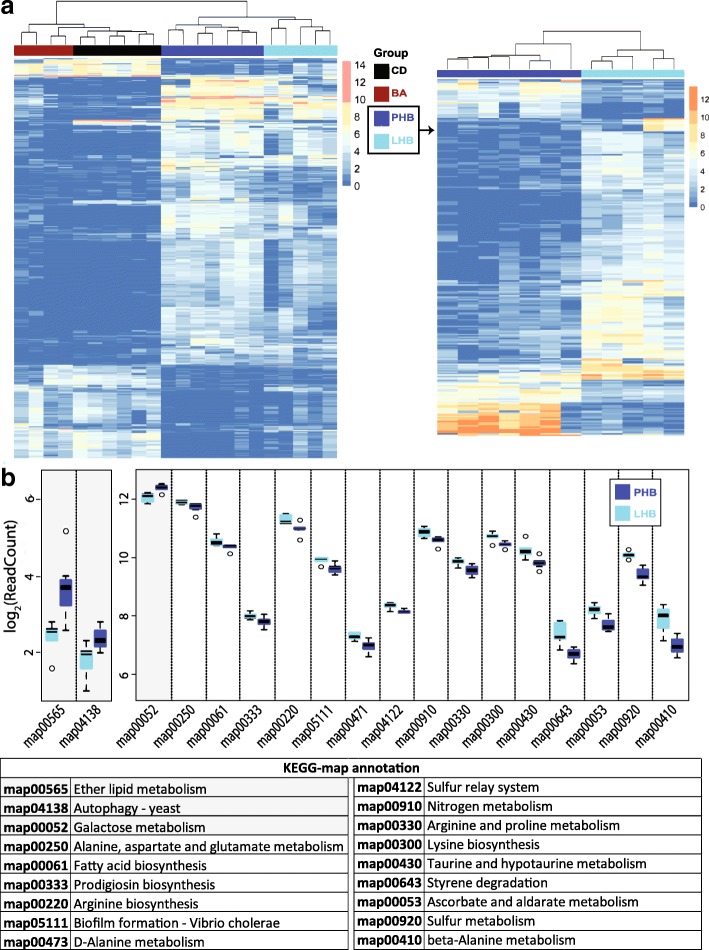
Diet-induced shifts in the metatranscriptome of mouse cecal microbiota. **a** Heat map of the 1207 genes with differential expression levels between the four diets. Genes were selected according to adjusted *p* values ≤ 0.001 and absolute(log2FC) ≥ 5. Mice were grouped into two main clusters corresponding to the BA/CD diets or the HFDs supplemented with BA. **b** Heat map depicting the expression of 266 genes showing differential expression level between the lard- and palm oil-based HFD. Genes were selected according to adjusted *p* values ≤ 0.001 and absolute(log2FC) ≥ 2.5. **c** Main metabolic pathways with significantly different expression level between the two HFDs

Cecal content from 22 fasted mice (CD, *n* = 7; BA, *n* = 4; PHB, *n* = 7; LHB, *n* = 5) were analyzed using microbial metatranscriptomics. On average, 14,906,345 ± 2,029,931 high-quality mRNA reads were obtained per mice and 2,424,413 ± 741,203 were mapped onto 180,412 ± 34,440 genes from the mouse metagenome catalog. Overall, the dietary interventions had a substantial impact on microbial activities: major clusters of mice according to microbial gene expression in the cecum were discriminated by HFD intake (Fig. 4a). Looking more specifically at differences between the two HFDs according to the metabolic phenotypes observed in mice, 266 genes were characterized by different levels of expression between LHB and PHB (Fig. 4b). Genes classified in the categories ether lipid metabolism (map00565), autophagy (map04138), and galactose metabolism (map00052) were overexpressed in mice fed palm oil compared with those fed lard (Fig. 4b). At the level of single KEGG Orthologies (KO) within the ether lipid metabolism pathway, two KOs were more prevalent in palm oil-fed mice: sucrose phosphorylase [K01058] and globoside alpha-N-acetylgalactosaminyltransferase (GBGT1) [K01114]. Among the top five differentially expressed genes, transcripts encoding enzymes linked to hyaluronic acid metabolism such as hyaluronate lyase [K01727] and hyaluronoglucosaminidase [K01197] were also more expressed (eight- and sevenfold, respectively) in palm oil-fed mice (Additional file 7: Table S2). On the other hand, 15 functional categories had a significantly higher expression in mice fed lard, including fatty acid biosynthesis (map00061), amino acid metabolism (alanine, aspartate, and glutamate metabolism, map00250; arginine biosynthesis, map00220; D-alanine metabolism, map00473; arginine and proline metabolism, map00330; lysine biosynthesis, map00300; taurine and hypotaurine metabolism, map00430; beta-alanine metabolism map00410), and sulfur metabolism (map00920) (Fig. 4c). In terms of KEGG Orthologies, glyceraldehyde 3-phosphate dehydrogenase (GAPDH) transcripts [K00134] were most highly regulated in lard-fed mice (ca. 4.5-fold overexpression) (Additional file 7: Table S2).

## Discussion

The major goal of the present study was to determine the functional implication of gut microbial populations for metabolic responses to bile acids and fat source in the diet of mice. Whereas germ-free mice appeared to be protected, colonized mice showed signs of metabolic disturbances when fat was provided as lard in a diet containing primary bile acids, which was accompanied by specific gut microbiota signatures.

### Gut microbial colonization and host metabolism

The fact that the body weight of germ-free mice was lower than that of colonized counterparts in our experiments is consistent with the literature [77]. We also demonstrate that the presence of gut microbes modulates hepatic lipid profiles: colonization was generally linked to elevated amounts of triglycerides and total fatty acids in the liver. Higher ratios of mono- to polyunsaturated fatty acids in colonized vs. germ-free animals suggest an increased fatty acid synthesis. It has been known for a while that the gut microbiota influences host lipid metabolism [77], but the interplay between gut microbes and dietary fat source has been highlighted more recently [9]. Our study confirms that the impact of diets containing fat of either animal or plant origin is dependent on intestinal microbial colonization. As reported by others [32], germ-free mice were characterized by high amounts of tauro-β-muricholic acid (TCDCA) even when feeding primary bile acids as in our study.

### Shifts in host metabolism and gut microbiota structure due to primary bile acid supplementation

Recently, Zheng et al. [78] reported that supplementation of bile acids alone in diet triggered metabolic disturbances similar to a HFD based on coconut oil (increased body weight, adipose tissue, hypercholesterolemia). Neither we nor Watanebe et al. [27] observed such an effect of bile acids. Methodological differences between the studies may explain this discrepancy, including the type and dose of bile acids supplemented to diets (e.g., 0.1% conjugated cholic acid in Zheng et al. vs. 0.2% free primary bile acids in our study), the genetic background of mice, or their age at feeding start (male C57BL/6J at 3 weeks of age vs. male C57BL/6N at 10 weeks of age). Moreover, Zheng et al. [78] reported mean relative abundances of 30 to 40% Proteobacteria in the cecum of mice on control diet (including members of the following various taxa: class *Campylobacterales*; family *Helicobacteraceae*; genus *Desulfovibrio*), which is rather unusual for laboratory mice and may also explain the different phenotypes observed [79, 80].

Only few studies have assessed gut microbiota changes induced by bile acids and findings seem to be study-dependent, most likely due to different experimental protocols and varying colonization status of mice at baseline. Islam et al. [40] investigated the impact of feeding approx. 0.2% cholic acid on the cecal microbiota of rats based on microscopic counts, clone libraries, and in situ hybridization. They reported decreased cell counts and Shannon diversity index whereas proportions of *Lachnospiraceae* and *Erysipelotrichaceae* were increased. In our study, primary bile acids alone reduced Shannon effective counts, but *Erysipelotrichaceae* were not detected at all in mice fed bile acids. Moreover, relative abundances of *Lachnospiraceae* were lower, except in the group fed palm oil. In another study, feeding 1% cholic acid to mice increased the density of bacterial populations capable of producing the secondary bile acid deoxycholic acid by 7-α-dehydroxylation, as determined in vitro using radioactively labelled substrate [81]. Via 16S rRNA amplicon sequencing, we did not find significant increase in the occurrence of known secondary bile acid-producing bacterial species, even though some of the yet uncultured species detected (e.g., dominant members of family S24-7) may be able to do so. Relative abundances of the family *Desulfovibrionaceae* (within the class *Deltaproteobacteria*) were increased in response to bile acid supplementation. In line with this finding, others found that relative abundances of *Desulfovibrionaceae*, which are Gram-negative sulphate-reducing bacteria, significantly increased in obese and metabolically impaired mice [82, 83].

### Impact of dietary fat sources on host metabolism

Published data showed that germ-free mice are per se not resistant to diet-induced obesity, i.e., their propensity to gain weight depends on the type of high-calorie diet used [8]. Kübeck et al. [9] recently reported that germ-free mice fed a lard-based HFD were resistant to diet-induced obesity partly due to increased energy expenditure, in contrast to mice fed a palm oil-based HFD. Interestingly, germ-free mice fed HFDs did not gain weight significantly in our experiments, neither based on lard nor palm oil and despite a feeding period similar to Kübeck et al. (8 weeks). This suggests that the addition of primary bile acids in the same HFD as in Kübeck et al. was sufficient to prevent obesity development in germ-free mice fed palm oil. This is in agreement with findings from 2006 by Watanabe et al. [27], who reported that a 7-week-long feeding of 0.5% (*w*/*w*) cholic acid to conventional C57BL/6J mice induced energy expenditure, which counteracted body weight gain induced by a high-fat diet. Even though information on fat source was not provided in this paper, colonized mice fed both a HFD and bile acids were as lean as control mice on a chow diet. In our experiments, however, this phenomenon was observed only in germ-free mice, which stayed lean, whereas conventional mice fed both bile acids and HFDs gained weight significantly when compared with mice on the control or BA diet. Additional experiments will be required to clarify whether the fat source in HFDs determines the possible anti-obesity effects of primary bile acids.

The gut-derived incretin hormones glucagon-like peptide 1 (GLP1) and glucose-dependent insulinotropic polypetide (GIP) are important factors determining glucose tolerance and insulin secretion from the pancreas. GIP and GLP1 show distinct expression patterns along the intestinal tract, GIP being produced in the proximal small intestine and GLP1 in distal parts of the small intestine and in the colon [84]. A subset of enteroendocrine cells (EEC), so-called L-cells, secretes GLP1 and their density was shown to be increased by dietary lipids both in mice and humans [85]. In the present study, quantifying the number of cells positive for GLP1 and the pan-EEC-marker chromogranin A in mouse colonic sections revealed that palm-based HFD feeding was associated with a significant increase in GLP1-producing EEC compared with all other diets, including lard-based HFD. Unchanged EEC numbers in the colon of LHB mice is in line with published data by Beyaz et al. [86] reporting no alteration in ChgA-positive cells in the jejunum of mice fed a 60%-kcal high-fat diet based on lard. Our results suggest that various dietary fat sources have different abilities to promote L-cell differentiation, the increased number of GLP1-producing EEC in the colon of PHB-fed mice possibly contributing to the improved glucose tolerance observed in these mice.

To the best of our knowledge, there is only one study that previously analyzed lipid profiles in the liver of GF and SPF mice fed different diets: Caesar et al. [87] investigated the impact of a lard-based or fish oil-based HFD fed to adult C57BL/6 mice for 11 weeks. The authors reported a dominant impact of diet compared with colonization status, which was not the case in our study, likely because bile acids were fed to mice in addition to HFDs. Nonetheless, the data by Caesar et al. support our finding that triglycerides and cholesteryl esters are elevated in the liver of mice fed lard. Decreased proportions of phosphatidylcholine in lard-fed mice characterized by detrimental metabolic responses are also in agreement with the literature [88].

### Impact of dietary fat sources on the gut microbiota

Amplicon sequencing of 16S rRNA genes from the cecal content of fasted SPF mice revealed diet-induced changes in gut microbiota diversity and composition. When comparing the two HFDs, PHB was linked to increased relative abundances of *Lachnospiraceae*, including one specific OTU with 92.6% similarity to *Acetatifactor muris*, a bacterium originally isolated from the cecum of an obese mouse [89]. This species is the closest relative to our OTU, yet at a sequence identity below genus-level thresholds. Other studies reported changes in the occurrence of *A*. *muris* relatives in the context of diet-induced obesity [9, 10, 67, 82]. The diversity and role of these bacteria in host metabolism will warrant further investigations. Two OTUs characterized by higher relative abundances following LHB feeding were identified at the species level as *Clostridium lactatifermentans* and *Flintibacter butyricus*. The former species is a lactate-fermenting bacterium producing the short-chain fatty acids acetate and propionate with traces of butyrate and isovalerate [90]. The latter species is capable of producing butyrate from amino acids [91], the metabolism of which seems to be affected by HFD as found in the present work by metatranscriptomics and in one of our previous study [92]. Nonetheless, no differences in colonic SCFA levels were observed in colonic content of the mice. The HFDs affected mouse cecal microbiota also at the functional level, as the metatranscriptomic approach identified genes and pathways affected by fat source. The expression of genes involved in ether lipid metabolism was similar between control and lard-fed mice but was significantly higher under palm oil feeding. Changes in ether lipid levels have been associated with host metabolic conditions, including nonalcoholic steatohepatitis, hypertension, obesity, and type-1 diabetes [93]. On the other hand, GAPDH transcript levels were higher in LHB vs. PHB mice; this gene and its activity were linked to obesity in rat models [94, 95]. Although speculative, these observations may partly explain the differential metabolic phenotypes observed in colonized mice fed palm oil vs. lard.

## Conclusions

We found that dietary fat source is an important factor that can substantially impact phenotypes in mouse models of diet-induced obesity. Lard in combination with primary bile acids in the diet had detrimental effects on the host metabolic state in colonized mice. The finding that germ-free mice were protected demonstrates the involvement of the gut microbiota, which was differentially affected at both the structural and functional level by the two high-fat diets.

## Additional files

Additional file 1: **Figure S1.** Experimental setup of the mouse trial. **a** Litter and cage distribution of mice used in the experiments. **b** Scheme of the experimental procedure. After a feeding period on control experimental diet (CD) between the age of 8 and 10 weeks for the sake of metabolic adaptation, GF and SPF mice were randomly divided into four different feeding groups (*n* = 9–12 per diet per colonization status): (I) CD; (II) CD supplemented with 0.2% (*w*/*w*) primary bile acids (BA); (III) palm oil-, or (IV) lard-based high-fat diet with 48 kJ% from fat, both supplemented with bile acids as above (P- and LHB, respectively). All diets were fed ad libitum for 8 weeks. At the end of the experimental feeding period, mice were divided into two groups prior to sampling: (I) fasted for 6 h and sacrificed immediately; (II) fasted for 6 h followed by oral glucose tolerance test (OGTT). (PNG 66 kb)

Additional file 2: **Figure S2.** Impact of experimental feedings and microbial colonization on mouse metabolism. **a** Body weight development over time. **b** Regression analysis of fasting blood insulin and leptin concentrations in P- and LHB-fed SPF mice. See the “Methods” section for description of statistical analyses. **c** Quantification of chromogranin A-positive (ChgA+) cells in colonic tissue sections of SPF mice from the different feeding groups. Description is as Fig. 1e. ***p < 0.01, one-way ANOVA followed by the Tukey test (performed using Graph Pad Prism). (PDF 9366 kb)

Additional file 3: **Figure S3.** Impact of experimental feedings and microbial colonization on the liver. **a** Liver to body weight ratio and corresponding regression analysis. **b** Liver histopathology. **c** Hepatic triglyceride concentrations. For detailed description of the statistical analysis see the “Methods” section. (PNG 576 kb)

Additional file 4: **Figure S4.** Colonization status of SPF and GF mice. **a** Cecum to body weight ratio. **b** Viable bacterial counts were determined by anaerobic cultivation. **C** 16S rRNA gene-targeted PCR of cecal content DNA from GF and SPF mice. Two representative samples per dietary group are shown for each colonization status. Bands at 1.5 kbp indicate the presence of microbes. Water was used as negative template control (NTC); number of mice: between 9 and 12 per group; for detailed description of the statistical analysis see the “Methods” section. (PNG 135 kb)

Additional file 5: **Table S1.** OTU-table based on high-throughput 16S rRNA amplicon analysis. Data were obtained and analyzed as described in the text. Data are sequence counts after quality checks. Only those OTUs occurrding at > 0.25% relative abundance in at least one sample were retained. Columns are individual mice per dietary groups as abbreviated in the text and in other illustrations. (PNG 78 kb)

Additional file 6: **Figure S5.** SCFA concentrations in colonic content of (XLSX 43 kb)

Additional file 7: **Table S2.** List of microbial genes differentially expressed in the cecum of mice fed high-fat diets supplemented with primary bile acids and with either palm oil (PHB) or lard (LHB) as fat source. Genes included in this table are significantly (q-value < 0.05) and substantially (>2.5-fold) overexpressed in one condition as compared to the other.Gene annotation (KEGG) refers to the Kyoto Encyclopedia of Genes and Genomes database annotation. (XLSX 34 kb)

## Abbreviations

12-DHCA: 12-Dihydroxycholic acid
AUC: Area under the curve
BA: CD supplemented with 0.1% (*w*/*w*) CA and 0.1% CDCA
C: Carbon
CA: Cholic acid
CD: Control diet
CE: Cholesteryl esters
CFU: Colony-forming unit
DCA: Deoxycholic acid
DTT: Dithiothreitol
EEC: Enteroendocrine cells
FXR: Farnesoid X receptor
GF: Germ-free
HFD: High-fat diet
LHB: Lard-based high-fat diet supplemented with 0.1% (*w*/*w*) CA and 0.1% CDCA
MCA: Muricholic acid
MUFA: Monounsaturated fatty acids
NTC: Negative template control
OGTT: Oral glucose tolerance test
OTU: Operational taxonomic units
PBS: Phosphate-buffered saline
PC: Phosphatidylcholine
PCA: Principal component analysis
PCR: Polymerase chain reaction
PHB: Palm oil-based high-fat diet supplemented with 0.1% (*w*/*w*) CA and 0.1% CDCA
PUFA: Polyunsaturated fatty acids
SAFA: Saturated fatty acids
SCFA: Short-chain fatty acids
SPF: Specific pathogen-free
TCDCA: Tauro-chenodeoxycholic acid
TGR5: G protein-coupled receptor
TLCA: Taurolithocholic acid
T-α-MCA: Tauro-α-muricholic acid
T-β-MCA: Tauro-β-muricholic acid
unsat: Unsaturated fatty acids
WAT: White adipose tissue
WCA: Wilkins-Chalgren-Anaerobe
