# Supplementary figures and images for: The gut microbiota drives the impact of bile acids and fat source in diet on mouse metabolism
Source: Microbiome. 2018 Aug 2;6:134. doi: 10.1186/s40168-018-0510-8 (PMC6091023; doi:10.1186/s40168-018-0510-8)

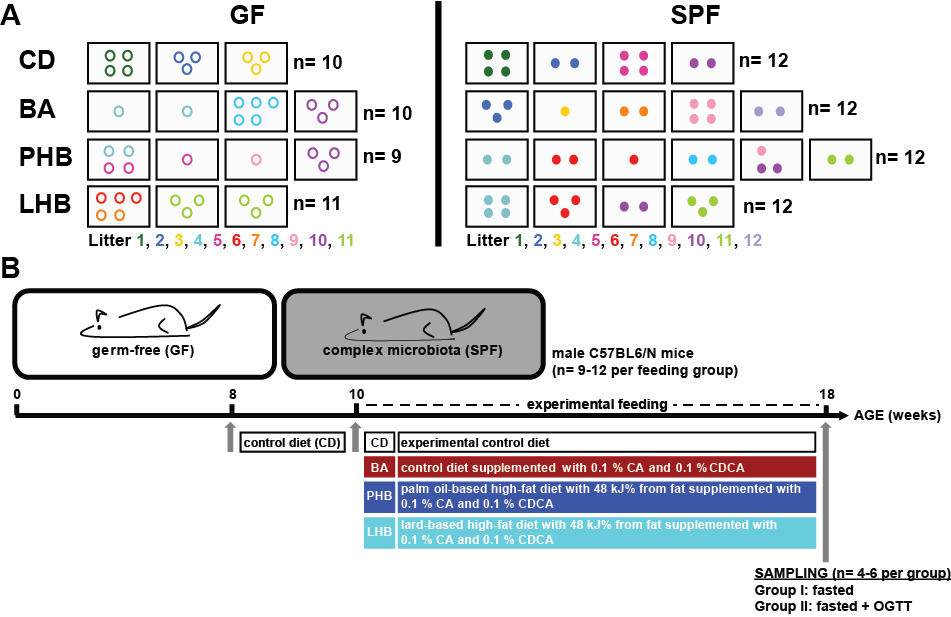

Supplement: Supplementary file 1 — Figure S1. Experimental setup of the mouse trial. a Litter and cage distribution of mice used in the experiments. b Scheme of the experimental procedure. After a feeding period on control experimental diet (CD) between the age of 8 and 10 weeks for the sake of metabolic adaptation, GF and SPF mice were randomly divided into four different feeding groups (n = 9–12 per diet per colonization status): (I) CD; (II) CD supplemented with 0.2% (w/w) primary bile acids (BA); (III) palm oil-, or (IV) lard-based high-fat diet with 48 kJ% from fat, both supplemented with bile acids as above (P- and LHB, respectively). All diets were fed ad libitum for 8 weeks. At the end of the experimental feeding period, mice were divided into two groups prior to sampling: (I) fasted for 6 h and sacrificed immediately; (II) fasted for 6 h followed by oral glucose tolerance test (OGTT). (PNG 66 kb) [file 40168_2018_510_MOESM1_ESM.png]

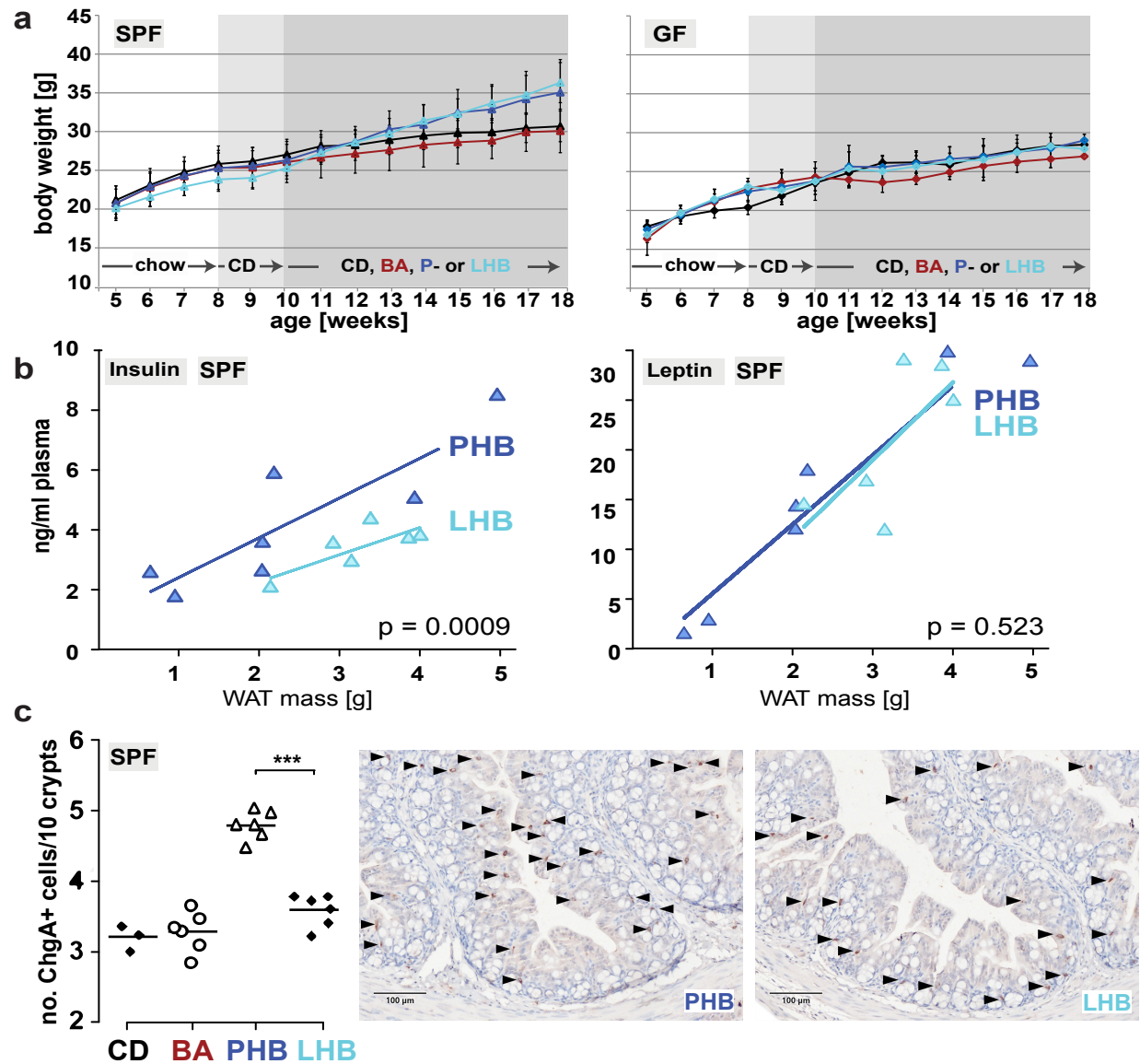

Supplement: Supplementary file 2 — Figure S2. Impact of experimental feedings and microbial colonization on mouse metabolism. a Body weight development over time. b Regression analysis of fasting blood insulin and leptin concentrations in P- and LHB-fed SPF mice. See the “Methods” section for description of statistical analyses. c Quantification of chromogranin A-positive (ChgA+) cells in colonic tissue sections of SPF mice from the different feeding groups. Description is as Fig. 1e. ***p < 0.01, one-way ANOVA followed by the Tukey test (performed using Graph Pad Prism). (PDF 9366 kb) [file 40168_2018_510_MOESM2_ESM.pdf]

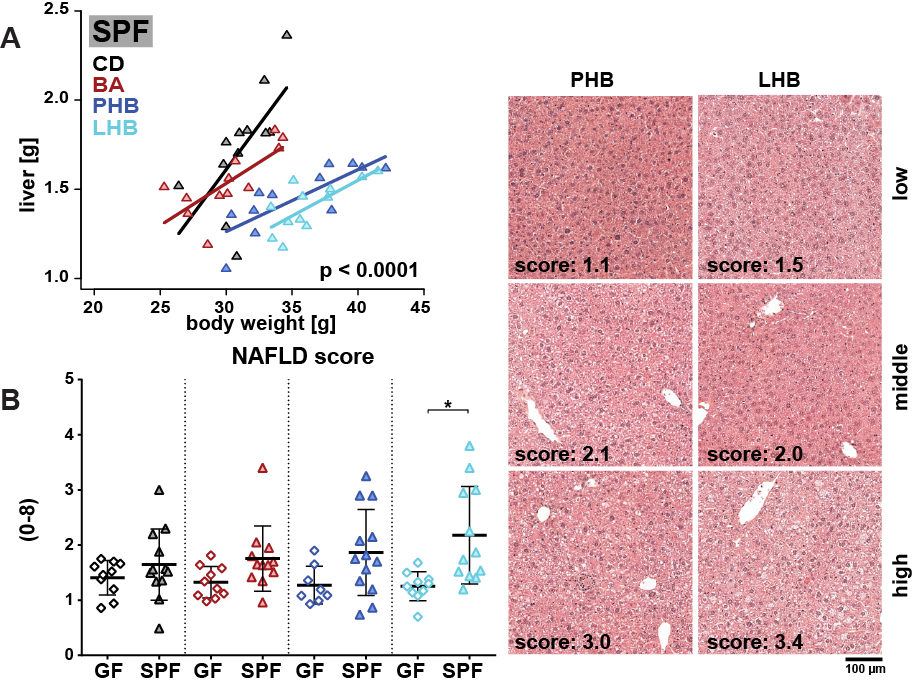

Supplement: Supplementary file 3 — Figure S3. Impact of experimental feedings and microbial colonization on the liver. a Liver to body weight ratio and corresponding regression analysis. b Liver histopathology. c Hepatic triglyceride concentrations. For detailed description of the statistical analysis see the “Methods” section. (PNG 576 kb) [file 40168_2018_510_MOESM3_ESM.png]

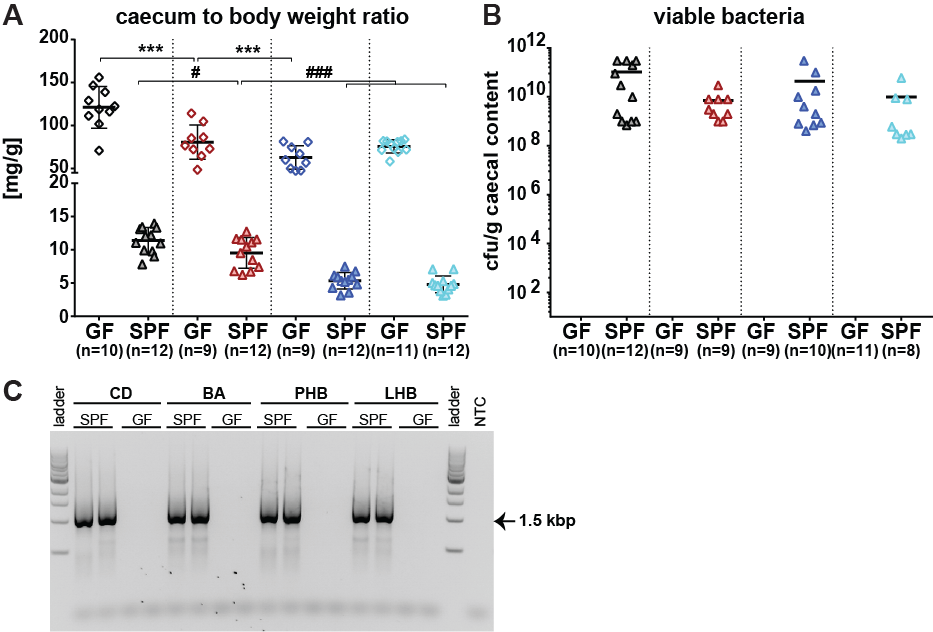

Supplement: Supplementary file 4 — Figure S4. Colonization status of SPF and GF mice. a Cecum to body weight ratio. b Viable bacterial counts were determined by anaerobic cultivation. C 16S rRNA gene-targeted PCR of cecal content DNA from GF and SPF mice. Two representative samples per dietary group are shown for each colonization status. Bands at 1.5 kbp indicate the presence of microbes. Water was used as negative template control (NTC); number of mice: between 9 and 12 per group; for detailed description of the statistical analysis see the “Methods” section. (PNG 135 kb) [file 40168_2018_510_MOESM4_ESM.png]

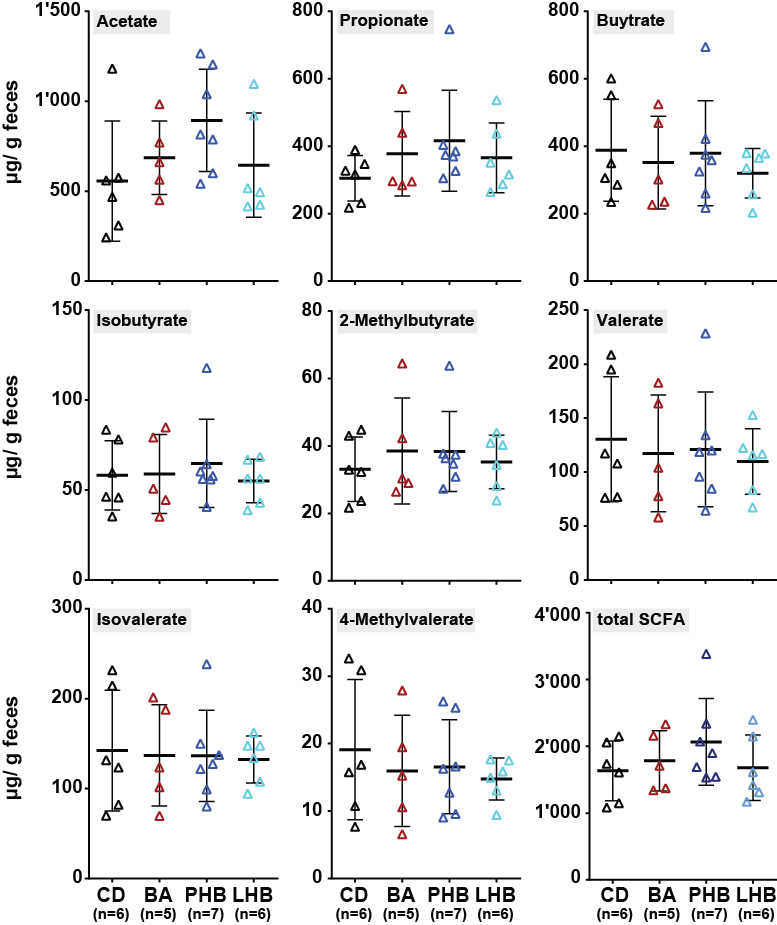

Supplement: Supplementary file 5 — Table S1. OTU-table based on high-throughput 16S rRNA amplicon analysis. Data were obtained and analyzed as described in the text. Data are sequence counts after quality checks. Only those OTUs occurrding at > 0.25% relative abundance in at least one sample were retained. Columns are individual mice per dietary groups as abbreviated in the text and in other illustrations. (PNG 78 kb) [file 40168_2018_510_MOESM5_ESM.png]
